# Reducing annotation burden in medical imaging with ADGNET: A semi-supervised deep learning strategy

**DOI:** 10.1371/journal.pone.0348596

**Published:** 2026-05-04

**Authors:** Xiaobo Yang

**Affiliations:** Department of Information Science and Technology, Zhejiang Shuren University, Hangzhou, Zhejiang, P. R.China; Eotvos Lorand University: Eotvos Lorand Tudomanyegyetem, HUNGARY

## Abstract

We propose ADGNET, a semi-supervised framework for Alzheimer’s disease (AD) diagnosis that jointly optimizes image reconstruction and classification through shared feature representations. The architecture integrates a residual backbone with attention modulation for dynamic feature selection, an encoder-decoder reconstruction branch for unsupervised representation learning, and a classification branch with focal loss to address class imbalance. This dual-task design enables effective feature learning from limited annotations. On two public MRI datasets—KACD (2D, 6,400 images) and ROAD (3D, 532 scans)—ADGNET achieves average performance improvements of 4.1% and 7.2% over state-of-the-art methods (ResNeXt WSL, SimCLR) across six metrics. Interpretability analysis using Grad-CAM and attention visualization confirms that the model focuses on clinically relevant neuroanatomical structures, particularly the hippocampus and temporal lobes, with strong correlation to established AD pathology (r = 0.67, p < 0.001). These results validate the model’s exceptional generalization capability and feature representation effectiveness across multi-modal medical imaging data, offering an efficient solution for few-shot medical image analysis.

## Introduction

Magnetic resonance imaging (MRI) is essential for Alzheimer’s disease (AD) diagnosis due to its sensitivity to brain structural changes [1]. While deep learning, particularly convolutional neural networks (CNNs), has advanced medical image analysis and AD grading [2], these models typically require large-scale expert-annotated datasets. The high cost and specialized expertise needed for medical image annotation create a critical bottleneck for clinical deployment.

Semi-supervised learning (SSL) offers a promising solution by leveraging abundant unlabeled data alongside limited labeled samples [3]. In computer vision, SSL has achieved remarkable success: ResNeXt WSL [4] demonstrated that pre-training on weakly-labeled Instagram images (940M) followed by fine-tuning on ImageNet achieves 85.4% Top-1 accuracy with minimal labels. Similarly, SimCLR [5] showed that contrastive learning enables high-precision classification (85.8% on ImageNet) using only 1% labeled data. These advances have inspired medical imaging applications, including multi-modal registration [6] and segmentation [7].

Despite these developments, existing SSL methods for medical imaging face two fundamental limitations: (1) they treat feature learning and task-specific objectives separately, missing synergies between unsupervised and supervised learning; and (2) they lack mechanisms to explicitly focus on disease-relevant anatomical regions critical for AD diagnosis.

To address these gaps, we propose ADGNET, a semi-supervised framework with three key innovations:

(1) Dual-task joint learning that simultaneously optimizes image reconstruction and classification through shared feature representations, enabling unsupervised feature learning to benefit the supervised task.
(2) Attention Modulation (AM) module that dynamically filters multi-scale features to enhance discriminative capacity and suppress irrelevant regions.
(3) Focal loss optimization specifically designed to handle class imbalance in medical datasets (e.g., rare moderate dementia cases).

While ADGNET shares the dual-task philosophy with existing semi-supervised frameworks, its architectural design and learning strategy present distinct characteristics. Unlike consistency-based SSL methods, which enforces prediction consistency between student and teacher models under different perturbations, ADGNET leverages a fundamentally different auxiliary task—image reconstruction—to learn meaningful feature representations from unlabeled data without requiring perturbation-based consistency regularization. Furthermore, compared to pseudo-labeling approaches that combine weak and strong augmentation with confidence-based pseudo-label generation, our method does not rely on thresholded pseudo-labels that may introduce confirmation bias, particularly under extreme data scarcity. Regarding U-Net-based co-training schemes [6], these typically employ shared encoder architectures for segmentation tasks with dual decoders or adversarial training. In contrast, ADGNET’s dual-branch design jointly optimizes a classification task (via fully connected layers with focal loss) and a reconstruction task (via a transposed convolutional decoder) from shared attention-modulated features. This explicit combination of (1) attention-modulated shared feature learning, (2) focal loss for imbalanced few-shot classification, and (3) reconstruction-based unsupervised pre-training followed by joint optimization represents a novel integration specifically tailored for few-shot medical image classification, distinguishing our approach from prior SSL paradigms that primarily focus on consistency regularization or pseudo-labeling strategies.

## ADGNET model architecture

The ADGNET model is a deep learning framework based on semi-supervised learning, utilizing a single-input multiple-output architecture. The model comprises two components: a backbone network equipped with an attention mechanism [8] and a dual-branch network. The backbone network employs a residual network structure, integrated with an attention modulation module (AM), allowing the overall framework to acquire effective feature representations while mitigating the impact of non-discriminative regions in the image. The dual-branch network is capable of executing two tasks in parallel: disease identification and image reconstruction. It harnesses the feature vectors produced by the backbone network, facilitating disease identification and image reconstruction via fully connected layers and decoder modules.

The backbone network designed in this study effectively mitigates the common gradient vanishing problem in deep model training through the incorporation of cross-layer connections. This network generates multi-scale feature pyramids, whose hierarchical features are utilized by two subsequent parallel sub-tasks, achieving parameter sharing and significantly improving computational efficiency. In the image reconstruction sub-task, this backbone network serves as an encoder to learn latent feature representations from images in an unsupervised manner, while in the disease identification sub-task, it functions as a feature extractor that updates parameters through supervised learning.

By incorporating the Attention Modulation (AM) module, each stage of the network can generate corresponding attention feature maps, as illustrated in (S1 Fig in S2 File).

As shown in S1 Fig in S2 File, given that the input feature map at the i-th layer has dimensions H × W × C, which corresponds to the output of the i-th stage in the ResNet architecture, the AM module generates channel attention weights denoted as CAF with dimensions 1 × 1 × C based on the input features. This enables the network to adaptively evaluate the importance of each feature channel, implementing a dynamic feature selection mechanism that enhances the model’s representational capacity while adding minimal computational overhead.

After obtaining the channel attention weights, an element-wise multiplication operation is applied to fuse the CAF with the original features F, resulting in a refined feature map F’. This process can be formally expressed as follows.


CAF = AM(F) (1)

$${\text{F}}^{\prime }\;=\text{ CAF }⊗\text{ F}$$ (2)

where AM denotes the Attention Modulation module and ⊗ represents the element-wise multiplication operator.

The dual-branch network consists of two sub-networks: a Classification Sub-Net (CSN) and a Reconstruction Sub-Net (RSN). Both sub-networks take the feature vector ${\text{V}}_{\text{f}}$ extracted by the backbone network as input. The CSN comprises a fully connected layer that maps the input features into a vector of dimension 1 × C. This vector is then processed through a Sigmoid activation function to produce a predicted probability vector ${\text{V}}_{\text{p}}$ with values ranging between 0 and 1, where each element represents the predicted probability for the corresponding disease category. The process of CSN can be expressed as follows.


$${\text{V}}_{\text{p}}=\sigma (\text{FC}({\text{V}}_{\text{f}}))$$ (3)

Here, FC denotes the fully connected operation, and σ represents the Sigmoid function.

The RSN adopts an encoder-decoder architecture, where the encoder consists of a backbone network and two fully connected (FC) layers named FCe1 and FCe2. The backbone network extracts and abstracts features, which are then encoded into a vector Ve through the two FC layers, completing the feature encoding process. The decoder comprises two components: a feature sampling section with multiple transposed convolutional layers, and a convolutional layer using the Tanh function [9] as activation. The input to the decoder is processed through two-dimensional planarization. Each transposed convolutional layer in the decoder contains multiple transposed convolutional kernels with a size of 3 × 3, stride of 2, and padding of 1. Through transposed convolutional layers and the ReLU function [10], the decoder decodes and samples the input feature maps. Utilizing convolutional layers and the Tanh function, it transforms the features into outputs matching the size of the input MRI image. Tanh is used here because the decoder’s output is not constrained to [0,1] after z-score normalization of the input images. While the input MRI intensities were normalized to zero mean and unit variance (z-score), the reconstruction target retains both positive and negative values. Tanh provides a symmetric output range [−1, 1] that matches this support, unlike sigmoid which is restricted to [0,1]. This alignment between activation range and target distribution facilitates training stability. The RSN process can be described as follows:


$${\text{V}}_{\text{e}}=\text{F}{\text{C}}_{\text{e2}}(\text{F}{\text{C}}_{\text{e1}}({\text{V}}_{\text{f}}))$$ (4)

$${\text{IM}}_{\text{r}}=\text{Tanh}(\text{Dec}({\text{V}}_{\text{e}}))$$ (5)

Here, $\text{F}{\text{C}}_{\text{e1}}$ and $\text{F}{\text{C}}_{\text{e2}}$ denote two fully connected layers, Dec represents the decoder, and ${\text{IM}}_{\text{r}}$ stands for the reconstructed image.

The ADGNET model optimizes its parameters based on a weighted loss function, which consists of two components and is defined as follows:


$$\text{L}=\lambda_{\text{1}}{\text{L}}_{\text{cls}}+\lambda_{\text{2}}{\text{L}}_{\text{rec}}$$ (6)

Among these, L denotes the total loss function, where ${\text{L}}_{\text{cls}}$ represents the classification loss, ${\text{L}}_{\text{rec}}$ corresponds to the reconstruction loss, and $\lambda_{\text{1}}$ and $\lambda_{\text{2}}$ are weighting factors that balance the contributions of these two loss components. The training of ADGNET follows a two-stage procedure. In the first stage (unsupervised pre-training), the model optimizes only the reconstruction loss Lrec using all available images (both labeled and unlabeled) from the training set. The backbone network and Reconstruction Sub-Net (RSN) learn to extract and decode meaningful feature representations without requiring annotations. In the second stage (supervised joint optimization), the pre-trained weights are used for initialization, and both the classification loss Lcls and reconstruction loss Lrec are jointly minimized using only the labeled subset. The total loss L = λ₁L_cls_ + λ₂L_rec_ simultaneously updates the shared backbone, the Classification Sub-Net (CSN), and the Reconstruction Sub-Net (RSN), enabling the reconstruction task to act as a regularizer that mitigates overfitting to scarce labeled data.

In designing the classification loss function ${\text{L}}_{\text{cls}}$, a focal approach is adopted by introducing a focal modification to the cross-entropy loss, which is defined as follows:


$${\text{L}}_{\text{cls}}=-\frac{\text{1}}{\text{N}}\sum_{\text{i}=\text{1}}^{\text{N}}\left({\left(\text{1}-{\text{P}}_{\text{i}}\right)}^{\gamma_{\text{p}}}\right)\text{log}(\text{1}-\overset{\prime }{\underset{\text{i}}{\text{P}}})+{{\text{P}}_{\text{i}}}^{\gamma_{\text{p}}}\text{log}(\overset{\prime }{\underset{\text{i}}{\text{P}}}))$$ (7)

Here, N denotes the number of samples involved in the optimization, $\gamma_{\text{p}}$ represents the category weight decay factor used to enhance the feature representation performance of the model (with a default value of 2), ${\text{P}}_{\text{i}}$ is the binary ground-truth label (0 or 1) for class i in the one-hot encoded target vector, and $\overset{\prime }{\underset{\text{i}}{\text{P}}}$ is the predicted probability for that class.

The reconstruction loss function ${\text{L}}_{\text{rec}}$ incorporates the mean squared error concept in its design and is defined as follows:


$${\text{L}}_{\text{rec}}=-\frac{\text{1}}{\text{N}}\sum_{\text{i}=\text{1}}^{\text{N}}{\left(\text{Y}-{\text{Y}}_{\text{i}}\right)}^{\text{2}}$$ (8)

Here, N denotes the number of optimized samples, Y represents the ground truth of the input sample, and ${\text{Y}}_{\text{i}}$ corresponds to the predicted value of the reconstructed sample.

## Comparative experiments

This study evaluates the performance of the proposed ADGNET model on two brain imaging datasets. The first dataset is from the Alzheimer’s Disease Recognition Challenge (referred to as ROAD) hosted by the China Computer Federation [11], while the second dataset is obtained from the publicly available Kaggle Alzheimer’s Classification Dataset (abbreviated as KACD) [12]. These datasets differ in terms of data scale, modality, and task design. The KACD dataset provides 6,400 two-dimensional MRI cases with one image per case, classified into four categories based on cognitive impairment severity: non-demented, very mildly demented, mildly demented, and moderately demented. In contrast, the ROAD dataset contains 532 cases of three-dimensional MRI images with a simplified classification task that only distinguishes between non-demented, mildly demented, and Alzheimer’s disease.

Both datasets underwent standardized preprocessing protocols to ensure consistency and reproducibility.

KACD dataset (2D MRI): The original 3D volumes from this publicly available dataset were preprocessed by the dataset providers. The pipeline included: (1) skull stripping using the Brain Extraction Tool (BET); (2) linear registration (affine) to the MNI152 standard space; (3) resizing to 128 × 128 pixels; (4) z-score intensity normalization (zero mean, unit variance) applied per image; and (5) selection of mid-axial slices containing key anatomical structures associated with Alzheimer’s disease (hippocampus, lateral ventricles).

ROAD dataset (3D MRI): This CCF challenge dataset was provided with comprehensive preprocessing completed. The pipeline included: (1) skull stripping using BET; (2) bias field correction to correct intensity inhomogeneities; (3) non-linear registration to the MNI152 template; (4) resampling to 1 mm³ isotropic resolution; (5) cropping to 128 × 128 × 128 voxels; and (6) global intensity normalization to the [0,1] range. All images were used in their preprocessed form without additional modification to maintain consistency with the original dataset specifications and challenge protocols.

We acknowledge that resizing the already-cropped images introduces interpolation artifacts. This step was necessary to match the fixed input dimensions required by the standard ResNet-50 (224 × 224) and 3D ResNet-50 (160 × 160 × 192) backbones, which were pre-initialized with weights from prior medical imaging studies. Third-order spline interpolation was used to minimize information loss.

Both datasets were pre-divided into training-validation sets (TVS) and independent test sets (TS). The KACD dataset contains 5,121 images in TVS and 1,279 images in TS, while the ROAD dataset comprises 300 and 232 3D MRI scans in its TVS and TS respectively. For experimental design, TVS was further subdivided into five parts through stratified sampling for internal cross-validation to tune model parameters. The independent TS was kept completely unseen during the entire training and validation process and was used only for the final performance evaluation of the best-selected model. Consequently, all reported metrics in this study represent the model’s performance on these fully held-out test sets.

To provide context for our methodological choices, we conducted a detailed analysis of class distributions in both datasets. The KACD dataset exhibits significant class imbalance, with the ‘moderately demented’ class comprising only 2.5% of samples (129 out of 5,121), while the ‘non-demented’ class represents 62.5% (3,200 samples). Similarly, the ROAD dataset shows moderate imbalance, with the ‘Alzheimer’s disease’ class accounting for 20% (60 out of 300 samples).

The focal loss parameter γ_p_ = 2 was selected based on systematic validation experiments comparing γ_p_ values from 0 to 5. γ_p_ = 2 achieved optimal performance on both datasets (91.2% accuracy on KACD and 89.8% on ROAD), effectively balancing the focus on hard examples from minority classes while maintaining learning efficiency on majority classes. For the severely imbalanced KACD dataset, we further augmented focal loss with class-specific weights (inversely proportional to class frequencies) to ensure adequate representation of the ‘moderately demented’ class during training.

The focal loss function introduces a modulating factor (1-Pi)^γ_p_ to the standard cross-entropy loss, where γ_p_ (gamma) controls the rate at which easy examples are down-weighted. When γ_p_ = 0, focal loss reduces to standard cross-entropy. As γ_p_ increases, the model focuses more on hard, misclassified examples while reducing the contribution of easy, well-classified samples. We set γ_p_ = 2 based on empirical validation, which provides an optimal balance:

- For a well-classified sample (Pi’ close to 1), (1-Pi)^γ_p_ ≈ 0, minimizing its loss contribution
- For a misclassified sample (Pi’ close to 0), (1-Pi)^γ_p_ ≈ 1, preserving its loss contribution

This mechanism is particularly valuable for our imbalanced medical datasets, ensuring that rare but clinically critical cases (e.g., moderate dementia, comprising only 2.5% of KACD) receive adequate learning attention.

To ensure reproducibility of our results, we provide comprehensive implementation details for both training phases. All experiments were conducted using PyTorch 1.9.0 on a platform with four NVIDIA GTX 2080Ti GPUs.

Data Preprocessing: For the KACD 2D dataset, images were resized to 224 × 224 pixels, normalized to zero mean and unit variance, and augmented with random horizontal flips (p = 0.5), random rotations (±10°), and random brightness/contrast adjustments. For the ROAD 3D dataset, volumes were resampled to 1 mm isotropic resolution and cropped to 160 × 160 × 192 voxels. The dataset was provided with intensity normalization to [0,1]; we used this normalization as-is without additional z-scoring. Only the KACD dataset was z-scored (zero mean, unit variance) following its original preprocessing pipeline.

Training Protocol: The training comprised two phases. In Phase 1 (unsupervised reconstruction pre-training), we used the Adam optimizer (β₁ = 0.9, β₂ = 0.999) with an initial learning rate of 1 × 10 ⁻ ³ for KACD and 5 × 10 ⁻ ⁴ for ROAD, applying cosine annealing decay. Batch sizes were 64 (KACD) and 16 (ROAD) with data parallelism across four GPUs. Training ran for 200 epochs (KACD) and 300 epochs (ROAD) with weight decay of 1 × 10 ⁻ ⁴ and gradient clipping at norm 1.0.

In Phase 2 (supervised classification fine-tuning), we used only 20% of labeled data. The AdamW optimizer was employed with initial learning rates of 1 × 10 ⁻ ⁴ (KACD) and 5 × 10 ⁻ ⁵ (ROAD), reduced on plateau (patience = 5, factor = 0.5). Batch sizes were 32 (KACD) and 8 (ROAD), with dropout rate 0.5 in FC layers. Focal loss with γ = 2.0 was applied, with additional class weights [0.4, 1.1, 2.0, 9.7] for the imbalanced KACD dataset. Training ran for 100 epochs (KACD) and 150 epochs (ROAD) with early stopping patience of 15.

Model Configuration: The backbone used ResNet-50 (2D) and ResNet-3D-50 (3D) with attention modulation modules (reduction ratio r = 16). The classification branch used a single FC layer (2048 → 4 or 2048 → 3) with sigmoid activation. The reconstruction branch employed a decoder with four transposed convolutional layers (kernel 3 × 3, stride 2) and a final Tanh-activated convolutional layer.

Model Selection: Validation was performed every epoch, with the best model selected based on validation accuracy. Final results reported are from a single evaluation on the completely held-out test sets using the best checkpoint. Total training time was approximately 10 hours for KACD and 19 hours for ROAD.

The model training process consisted of two phases: first, an unsupervised pre-training phase where the reconstruction sub-network and shared feature layers were trained using all images in TVS (labeled and unlabeled) to minimize reconstruction loss only; subsequently, a supervised joint optimization phase was conducted using only 20% of the labeled data from TVS, where both classification and reconstruction losses were jointly minimized to fine-tune the entire network. The final evaluation of model performance was independently performed on TS which did not participate in the training process.

To comprehensively evaluate the performance of the ADGNET model, this study employs six distinct evaluation metrics: Kappa coefficient (Kappa), sensitivity (Sen), specificity (Spe), precision (Pr), accuracy (Acc), and F1-Score (F1). These metrics assess the model from multiple dimensions including prediction consistency, positive identification capability, negative identification capability, predictive reliability, and overall performance.

The calculation of each metric is based on four fundamental elements in classification results: true positives (TP), true negatives (TN), false positives (FP), and false negatives (FN). The specific computational formulas are as follows:

Kappa coefficient: This metric measures the agreement between model predictions and ground truth values, calculated based on the observed accuracy (po) and expected accuracy (pe). The computational formulas for these two accuracy metrics are as follows:


po = (TP + TN) / N (9)

pe = [(TN+FN)(TN+FP) + (TP+FP)(TP+FN)] / (N*N) (10)

The formula for calculating the Kappa coefficient is as follows:


Kappa = (po − pe) / (1 − pe) (11)

Sensitivity (Sen): This metric reflects the model’s ability to correctly identify positive samples, and its calculation formula is as follows:


Sen = TP / (TP + FN) (12)

Specificity (Spe): This metric quantifies the model’s capability to correctly identify negative samples, with its computational formula expressed as follows:


Spe = TN / (TN + FP) (13)

Precision (Pr): This metric measures the proportion of true positive cases among all samples predicted as positive by the model. The computational formula is defined as follows:


Pr = TP / (TP + FP) (14)

Accuracy (Acc): This metric represents the proportion of correct predictions among all predictions made, and its computational formula is defined as follows:


Acc = (TP + TN) / (TP + TN + FP + FN) (15)

F1-Score: This metric represents the harmonic mean of precision and sensitivity, providing a comprehensive evaluation of the model’s robustness. The computational formula is defined as follows:


F1−Score = 2 * Pr * Sen / (Pr + Sen) (16)

This study employs the aforementioned metrics to conduct a comprehensive comparative evaluation of the proposed ADGNET model against other state-of-the-art semi-supervised learning methods.

Using brain MRI datasets comprising both 2D and 3D structures, we systematically validate the proposed semi-supervised learning framework ADGNET. To thoroughly assess model performance, mainstream semi-supervised learning approaches—including ResNext WSL [13] and SimCLR [14]—are selected for comparative experiments with our method. Quantitative analysis is performed using six metrics: Kappa, Sensitivity (Sen), Specificity (Spe), Precision (Pr), Accuracy (Acc), and F1-Score, thereby examining the generalization capability, transfer-ability, and overall effectiveness of ADGNET. All experimental codes are implemented based on the PyTorch framework, with model training and testing conducted on a computational platform equipped with four NVIDIA GTX 2080Ti GPUs and an Intel Xeon E5-2600 v4 3.60GHz CPU.

The model training process consists of two sub-processes: an image reconstruction task training sub-process and a disease identification task training sub-process, as illustrated in (S2 Fig in S2 File).

As shown in S2 Fig in S2 File, the entire process embodies a typical semi-supervised learning paradigm: it first learns general feature representations through an unsupervised task (reconstruction), followed by fine-tuning via a supervised task (classification), ultimately yielding a high-performance disease classification model.

To comprehensively evaluate ADGNET, we compare against six state-of-the-art semi-supervised learning methods spanning different SSL paradigms. In addition to the self-supervised pre-training approaches ResNeXt WSL and SimCLR, we include four SSL frameworks specifically designed for few-shot classification and successfully adapted to medical imaging tasks: (1) Mean Teacher [1,7], which employs an exponential moving average teacher to generate consistent pseudo-labels; (2) FixMatch [2,15], combining weak-to-strong augmentation consistency with confidence-based pseudo-labeling; (3) MixMatch [3,16], integrating mixup augmentation with entropy minimization; and (4) MoCo-v2 [5,6], a momentum contrastive learning framework adapted for MRI analysis.

All methods are implemented using identical backbone architectures (ResNet-50 for 2D, ResNet-3D-50 for 3D) and trained under the same data partition protocol. Hyperparameters for each baseline are optimized according to their original publications, with additional validation on our datasets to ensure fair comparison. The evaluation covers multiple performance metrics, with all results calculated with 95% confidence intervals to ensure assessment accuracy and statistical reliability. The results are presented in (S3 Fig in S2 File).

As shown in S3 Fig in S2 File, the ADGNET model outperforms the other two methods across all six evaluation metrics, with average metric values exceeding those of ResNext WSL and SimCLR by 4.1% and 1.4% respectively. This demonstrates that ADGNET exhibits smaller performance fluctuations and higher overall recognition accuracy in Alzheimer’s disease identification tasks based on 2D brain MRI images.

Furthermore, to further validate the superiority of the ADGNET model, we conducted comparative performance evaluations against two state-of-the-art semi-supervised methods—ResNext WSL and SimCLR—on the ROAD 3D MRI test dataset. All methods were comprehensively compared based on six evaluation metrics, with results presented using 95% confidence intervals to ensure statistical rigor and reliability of the assessment. The results are shown in (S4 Fig in S2 File).

As shown in S4 Fig in S2 File, the ADGNET model demonstrates superior performance with average metric values exceeding ResNext WSL and SimCLR by 7.2% and 5.4% respectively across all six evaluation metrics. This result reaffirms that ADGNET achieves higher performance than the two mainstream semi-supervised learning methods in 3D MRI image analysis tasks, indicating its enhanced generalization and transfer capabilities across diverse medical imaging datasets.

To rigorously validate the performance improvements of ADGNET over baseline methods, we conducted comprehensive statistical significance testing. For each dataset and evaluation metric, we performed paired comparisons using both parametric (paired t-test) and non-parametric (Wilcoxon signed-rank test) methods across the 5 cross-validation folds. To control for Type I errors due to multiple comparisons, we applied Bonferroni correction (adjusted α = 0.05/6 ≈ 0.0083 for six metrics).

All improvements of ADGNET over baseline methods achieved statistical significance (p < 0.01 after correction) across both datasets. Effect sizes (Cohen’s d) ranged from 0.78 (medium effect) to 1.42 (large effect), indicating that the observed improvements are not only statistically significant but also practically meaningful. The consistency between parametric and non-parametric test results confirms the robustness of our findings regardless of distributional assumptions.

## Conclusions

Addressing the practical challenge of scarce annotated medical imaging data, this paper proposes ADGNET—a semi-supervised model based on dual-task joint learning—which demonstrates remarkable effectiveness in Alzheimer’s disease (AD) classification tasks. The main conclusions and future perspectives are summarized as follows:

(1) Our results demonstrate that joint optimization of reconstruction and classification within a shared attention-modulated backbone offers distinct advantages over consistency-based SSL methods (e.g., Mean Teacher, FixMatch) and contrastive learning approaches (SimCLR, MoCo-v2). Unlike consistency regularization, which relies on perturbation invariance — often fragile in medical images with large anatomical variation — ADGNET’s reconstruction task provides a direct supervisory signal for learning structural features from unlabeled data. This is particularly beneficial when labeled data are extremely scarce (20% in our experiments).
(2) The attention modulation module consistently improved performance across both datasets, with ablation studies (now included in Supporting Information) showing that removing AM reduced accuracy by 3.2% on KACD and 4.5% on ROAD. This confirms that dynamically filtering channel-wise features helps suppress irrelevant regions (e.g., background, skull remnants) while enhancing disease-relevant structures.
(3) Several limitations should be acknowledged. First, our two-stage training doubles computational cost compared to single-stage SSL methods. Second, the model was evaluated only on AD diagnosis; generalization to other diseases (e.g., Parkinson’s, brain tumors) remains untested. Third, the datasets, though public, come from limited institutions; multi-center validation is needed. Fourth, the reconstruction target (original image) may encourage encoding of irrelevant details (e.g., noise, skull edges) — future work could adopt perceptual or contrastive reconstruction losses.

## Appendix

We have created a public GitHub repository containing the complete ADGNET implementation:

Repository: https://github.com/[anonymous]/ADGNET (anonymized for blind review; the actual repository will be made public upon acceptance)

The repository includes:

- Full model architecture code (backbone, AM module, dual branches)
- Training scripts for both phases
- Data preprocessing pipelines for KACD and ROAD datasets
- Evaluation scripts with all six metrics
- Visualization tools (Grad-CAM, attention maps)
- Pretrained model weights via Zenodo link
- Docker container for environment reproduction
- Detailed README with installation and usage instructions

## Supporting information

S1 File Supporting Information – Lab Data-v4. (DOCX)

S2 File Supporting Information-Figure-PLOS ONE-v4. (DOCX)
