# Supplementary material for: Reducing annotation burden in medical imaging with ADGNET: A semi-supervised deep learning strategy
Source: PLoS One. 2026 May 4;21(5):e0348596. doi: 10.1371/journal.pone.0348596 (PMC13138640; doi:10.1371/journal.pone.0348596)
Supplement: S1 File — (DOCX) [file pone.0348596.s001.docx]

Supporting Information

**Performance Comparison of Seven Methods on the KACD Dataset**

| algorithm |  | | | | | |
| --- | --- | --- | --- | --- | --- | --- |
| ResNext WSL | Six evaluation metrics | | | | | |
|  | Kappa | Sen | Spe | Pr | Acc | F1-Score |
|  | Performance indicator value | | | | | |
|  | 0.9611 | 0.9712 | 0.9701 | 0.9684 | 0.9615 | 0.9537 |
| SimCLR | Six evaluation metrics | | | | | |
|  | Kappa | Sen | Spe | Pr | Acc | F1-Score |
|  | Performance indicator value | | | | | |
|  | 0.9745 | 0.9875 | 0.9859 | 0.9860 | 0.9765 | 0.9789 |
| Mean Teacher | Six evaluation metrics | | | | | |
|  | Kappa | Sen | Spe | Pr | Acc | F1-Score |
|  | Performance indicator value | | | | | |
|  | 0.9501 | 0.9602 | 0.9504 | 0.9404 | 0.9305 | 0.9307 |
| FixMatch | Six evaluation metrics | | | | | |
|  | Kappa | Sen | Spe | Pr | Acc | F1-Score |
|  | Performance indicator value | | | | | |
|  | 0.9402 | 0.9501 | 0.9401 | 0.9302 | 0.9208 | 0.9201 |
| MixMatch | Six evaluation metrics | | | | | |
|  | Kappa | Sen | Spe | Pr | Acc | F1-Score |
|  | Performance indicator value | | | | | |
|  | 0.9301 | 0.9402 | 0.9303 | 0.9201 | 0.9102 | 0.9103 |
| MoCo-v2 | Six evaluation metrics | | | | | |
|  | Kappa | Sen | Spe | Pr | Acc | F1-Score |
|  | Performance indicator value | | | | | |
|  | 0.9131 | 0.9301 | 0.9143 | 0.9101 | 0.8805 | 0.8723 |
| ADGNET method | Six evaluation metrics | | | | | |
|  | Kappa | Sen | Spe | Pr | Acc | F1-Score |
|  | Performance indicator value | | | | | |
|  | 0.9922 | 0.9969 | 0.9953 | 0.9955 | 0.9961 | 0.9978 |

**Performance Comparison of Seven Methods on the ROAD Dataset**

| algorithm |  | | | | | |
| --- | --- | --- | --- | --- | --- | --- |
| ResNext WSL | Six evaluation metrics | | | | | |
|  | Kappa | Sen | Spe | Pr | Acc | F1-Score |
|  | Performance indicator value | | | | | |
|  | 0.8817 | 0.9301 | 0.9311 | 0.9256 | 0.9215 | 0.9211 |
| SimCLR | Six evaluation metrics | | | | | |
|  | Kappa | Sen | Spe | Pr | Acc | F1-Score |
|  | Performance indicator value | | | | | |
|  | 0.9270 | 0.9441 | 0.9485 | 0.9331 | 0.9356 | 0.9451 |
| Mean Teacher | Six evaluation metrics | | | | | |
|  | Kappa | Sen | Spe | Pr | Acc | F1-Score |
|  | Performance indicator value | | | | | |
|  | 0.8511 | 0.9201 | 0.9204 | 0.9011 | 0.9105 | 0.9101 |
| FixMatch | Six evaluation metrics | | | | | |
|  | Kappa | Sen | Spe | Pr | Acc | F1-Score |
|  | Performance indicator value | | | | | |
|  | 0.8402 | 0.9103 | 0.9102 | 0.9002 | 0.9011 | 0.9008 |
| MixMatch | Six evaluation metrics | | | | | |
|  | Kappa | Sen | Spe | Pr | Acc | F1-Score |
|  | Performance indicator value | | | | | |
|  | 0.8301 | 0.8872 | 0.8901 | 0.8803 | 0.8901 | 0.8903 |
| MoCo-v2 | Six evaluation metrics | | | | | |
|  | Kappa | Sen | Spe | Pr | Acc | F1-Score |
|  | Performance indicator value | | | | | |
|  | 0.8151 | 0.8709 | 0.8802 | 0.8701 | 0.8755 | 0.8513 |
| ADGNET method | Six evaluation metrics | | | | | |
|  | Kappa | Sen | Spe | Pr | Acc | F1-Score |
|  | Performance indicator value | | | | | |
|  | 0.9736 | 0.9805 | 0.9927 | 0.9899 | 0.9871 | 0.9859 |
