# Supplementary material for: Reducing annotation burden in medical imaging with ADGNET: A semi-supervised deep learning strategy
Source: PLoS One. 2026 May 4;21(5):e0348596. doi: 10.1371/journal.pone.0348596 (PMC13138640; doi:10.1371/journal.pone.0348596)
Supplement: S2 File — (DOCX) [file pone.0348596.s002.docx]

Supporting Information: Figure

**S1 Fig. 1. Schematic diagram of attention module**

**S2 Fig. 2. Illustration of the Model Training Process**

**S3 Fig. 3. Performance Comparison of Seven Methods on the KACD Dataset**

**S4 Fig. 4. Performance Comparison of Seven Methods on the ROAD Dataset**
